# Establishment of the auxin inducible degron system for *Babesia duncani*: a conditional knockdown tool to study precise protein regulation in *Babesia* spp.

**DOI:** 10.1186/s13071-024-06458-4

**Published:** 2024-10-31

**Authors:** Bo Chen, Qi Zhang, Sen Wang, Xing-ai Guan, Wan-xin Luo, Dong-fang Li, Yue He, Shu-jing Huang, Ya-ting Zhou, Jun-long Zhao, Lan He

**Affiliations:** 1 State Key Laboratory of Agricultural Microbiology, College of Veterinary Medicine, Huazhong Agricultural University, Wuhan, 430070 Hubei China; 2 Key Laboratory of Preventive Veterinary Medicine in Hubei Province, Wuhan, 430070 Hubei China

**Keywords:** *Babesia duncani*, Conditional knockdown system, Auxin-inducible degron, Protein degradation

## Abstract

**Background:**

*Babesia duncani* is a pathogen within the phylum Apicomplexa that causes human babesiosis. It poses a significant threat to public health, as it can be transmitted not only through tick bites but also via blood transfusion. Consequently, an understanding of the gene functions of this pathogen is necessary for the development of drugs and vaccines. However, the absence of conditional gene knockdown tools has hindered the research on this pathogen. The auxin-inducible degron (AID) system is a rapid, reversible conditional knockdown system widely used in gene function studies. Thus, there is an urgent need to establish the AID system in *B. duncani* to study essential gene functions.

**Methods:**

The endogenous genes of the Skp1-Cullin-F-box (SCF) complex in *B. duncani* were identified and confirmed through multiple sequence alignment and conserved domain analysis. The expression of the F-box protein TIR1 from *Oryza sativa* (OsTIR1) was achieved by constructing a transgenic parasite strain using a homologous recombination strategy. Polymerase chain reaction (PCR), western blot, and indirect immunofluorescence assay (IFA) were used to confirm the correct monoclonal parasite strain. The degradation of enhanced green fluorescent protein (eGFP) tagged with an AID degron was detected through western blot and live-cell fluorescence microscopy after treatment of indole-3-acetic acid (IAA).

**Results:**

In this study, Skp1, Cul1, and Rbx1 of the SCF complex in *B. duncani* were identified through sequence alignment and domain analysis. A pure BdTIR1 strain with expression of the OsTIR1 gene was constructed through homologous recombination and confirmed. This strain showed no significant differences from the wild type (WT) in terms of growth rate and proportions of different parasite forms. The eGFP tagged with an AID degron was successfully induced for degradation using 500 μM IAA. Grayscale analysis of western blot indicated a 61.3% reduction in eGFP expression levels, while fluorescence intensity analysis showed a 77.5% decrease in fluorescence intensity. Increasing the IAA concentration to 2 mM accelerated eGFP degradation and enhanced the extent of degradation.

**Conclusions:**

This study demonstrated the functionality of the AID system in regulating protein levels by inducing rapid degradation of eGFP using IAA, providing an important research tool for studying essential gene functions related to invasion, egress, and virulence of *B. duncani*. Moreover, it also offers a construction strategy for apicomplexan parasites that have not developed an AID system.

**Graphical Abstract:**

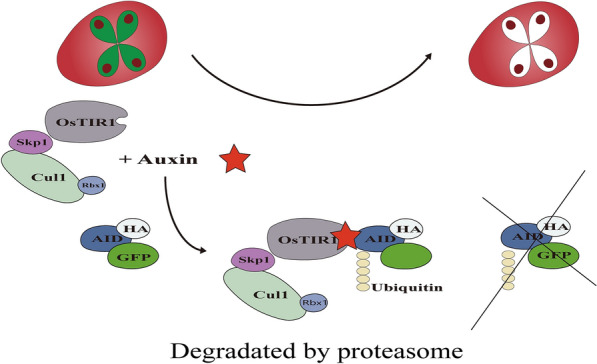

**Supplementary Information:**

The online version contains supplementary material available at 10.1186/s13071-024-06458-4.

## Background

Human babesiosis is a zoonotic parasitic disease caused by parasites of the genus *Babesia* within the phylum Apicomplexa [1]. The major causative pathogens of human babesiosis include *Babesia microti* [2], *B. divergens* [3], *B. venatorum* [4], *B. duncani* [1], *B. crassa*–like [5], *Babesia* sp. XXB/HangZhou [6], *Babesia* sp. KO-1 [7], and *B. divergens*-like [8]. Infection with these pathogens can lead to a malaria-like illness characterized by symptoms such as high fever, anemia, hemoglobinuria, and even death, particularly for immunocompromised individuals [9].

In recent years, *B. duncani* has raised increasing public health concerns, as it can be transmitted not only through tick bites but also through blood transfusion [10–12]. This pathogen is a recently identified zoonotic parasite, first discovered in the blood of a patient in Washington state, USA, in 1991 [13, 14]. Since its discovery, *B. duncani* has predominantly prevailed in the United States, and has shown a trend of global spread, with recent detections in ticks in South Korea [15]. Therefore, there is a need for comprehensive research on this pathogen to develop drugs and vaccines for the protection of human health.

Significant progress has been made in the study of *B. duncani*. An in vitro culture system for *B. duncani* using hamster red blood cells (RBC) was established as early as 1994 [16, 17], and the development of serum-free culture medium has reduced both the complexity and cost of research on this pathogen [18]. In addition to the mature in vitro cultivation system, the establishment of animal models has further improved the research methods for *B. duncani* [19, 20]. Transient and stable transfection systems using a homologous recombination strategy have been established, providing a genetic modification approach suitable for *B. duncani* [21]. A better understanding of the essential genes of *B. duncani* is of primary importance for the development of vaccines and drugs to prevent and control its infection. However, functional analysis of essential genes of *B. duncani* is hampered by the lack of a precise conditional protein modulation method.

**Fig. 1:**
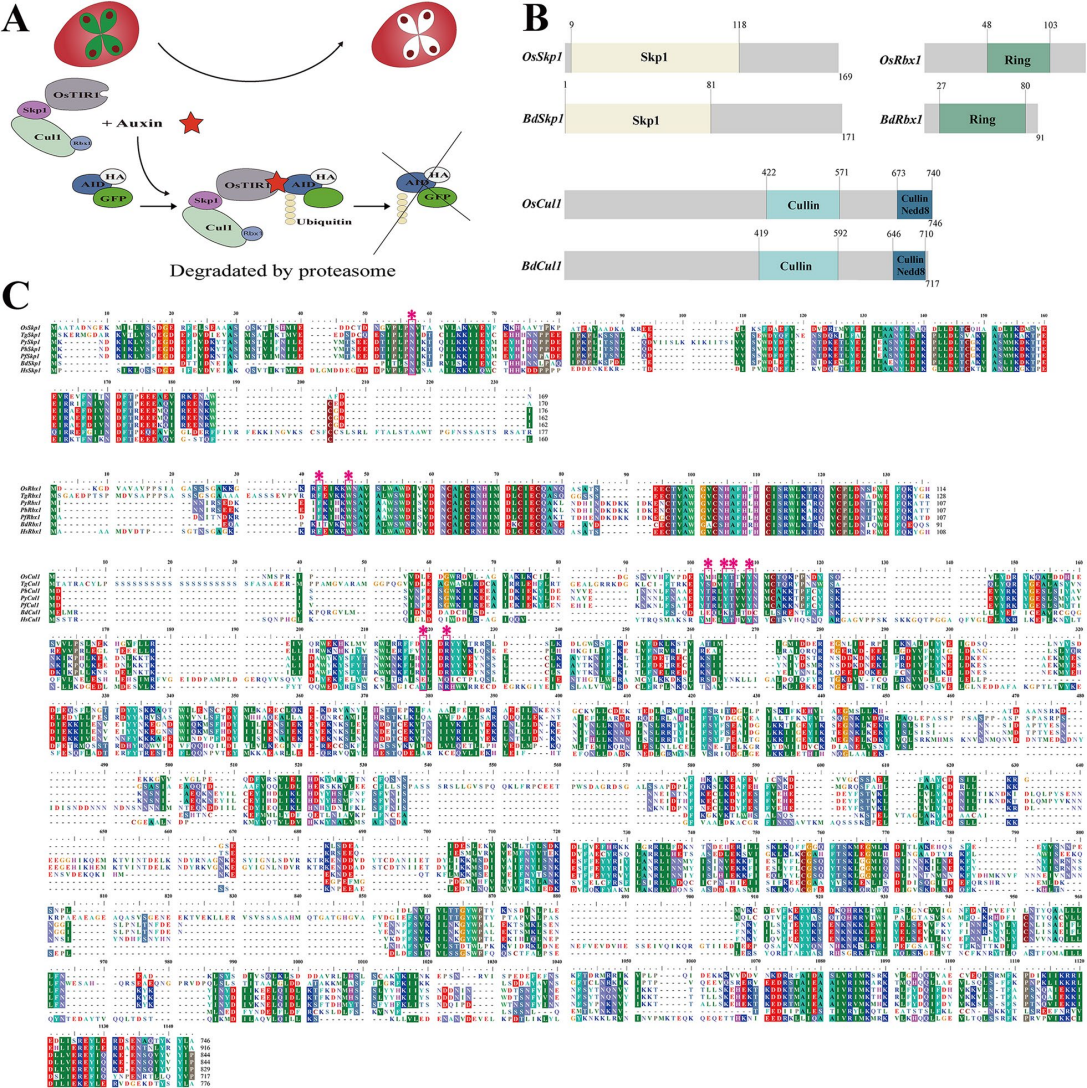
Conserved domains of the homologs of the SCF complex. **A** Schematic representation of the AID system adapted from Nishimura et al. [22]. OsTIR1 (an F-box protein) responds to auxin, leading to the recruitment of AID degron-tagged protein to the SCF complex, resulting in ubiquitination and degradation of the target protein. **B** Comparison of conserved domains of the homologs of SCF complexes between *O. sativa* and *B. duncani* by SMART analysis. **C** Sequence alignments of conserved domains of the SCF complex from *O. sativa*, *Homo sapiens*, *B. duncani*, and species that have an established AID system in the phylum Apicomplexa. The key amino acid residues based on crystal structures of *H. sapiens* SCF complex were labeled by * and box

The auxin-inducible degron (AID) system is a rapid and specific tool for generating mutants of essential proteins responsive to auxin, facilitating the study of gene function [22]. This system depends on the evolutionarily conserved eukaryotic SCF ubiquitin ligase complex, comprising the Skp1, Cul1, and Rbx1, which are conserved in eukaryotic cells, and the F-box is typically TIR1 from *Oryza sativa* (OsTIR1). However, the TIR1 and AUX/IAA (AID degron) homologous genes are only found in plant cells. Thus, functionalization of the AID system requires the expression of the OsTIR1 protein and AID degron in non-plant cells. The OsTIR1 protein responds to indole-3-acetic acid (IAA), initiating the ubiquitination of proteins of interest (POI) tagged with AID degron and subsequent degradation by the proteasome (Fig. 1A). The AID system has been successfully applied in apicomplexan parasites including *Plasmodium falciparum* [23], *Plasmodium berghei* [24], *Plasmodium yoelii* [25], and *Toxoplasma gondii* [26]. However, there is no report of the use of the AID system in *Babesia* spp.

In this study, Skp1, Rbx1, and Cul1 were identified and confirmed in *B. duncani*, and a transgenic parasite strain stably expressing the OsTIR1 protein was constructed, thereby facilitating the formation of a complete SCF complex. Using it as the parental strain, stable expression of the AID degron-tagged enhanced green fluorescent protein (eGFP) was achieved through homologous recombination for functional testing of the AID system. Successful induction of eGFP degradation was achieved using IAA, thereby demonstrating the function of protein degradation of the AID system in *B. duncani*. This study provides a conditional knockdown method for exploring potential drug targets and virulence genes in *B. duncani*, offering a construction strategy applicable to apicomplexan parasites lacking an established AID system.

## Methods

### Sequence alignment and conservative domain analysis

The genomic and protein information was obtained from the National Genomics Data Center of the China National Center for Bioinformation (CNCB, https://www.cncb.ac.cn/) under accession number GWHBECJ00000000 [27]. Multiple protein sequence alignment of the SCF complex was performed using Mafft-7.520 [28] and the Basic Local Alignment Search Tool (BLAST), while the conserved domains were predicted by the Simple Modular Architecture Research Tool (SMART) online service (http://smart.embl.de/) [29, 30].

### In vitro culture of *B. duncani*

Hamster RBCs were collected for the culture of *B. duncani* according to previously reported procedures [18, 21]. *Babesia duncani* strain WA1 (ATCC PRA-302™) was obtained from the American Type Culture Collection (ATCC) and maintained in our laboratory (State Key Laboratory of Agricultural Microbiology, College of Veterinary Medicine, Huazhong Agricultural University, China). *Babesia duncani* was cultured in vitro [31] in serum-free medium [18] under microaerophilic conditions (5% CO_2_, 2% O_2_, and 93% N_2_) at 37 °C. VP-SFM AGT™ (Gibco Life Technologies, Shanghai, China) was used as basal medium, supplemented with 2 mg/ml AlbuMax™ I (Gibco Life Technologies, Shanghai, China), 200 μM l-glutamine (Sigma-Aldrich, Shanghai, China), and antibiotics.

### Plasmid construction

**Fig. 2:**
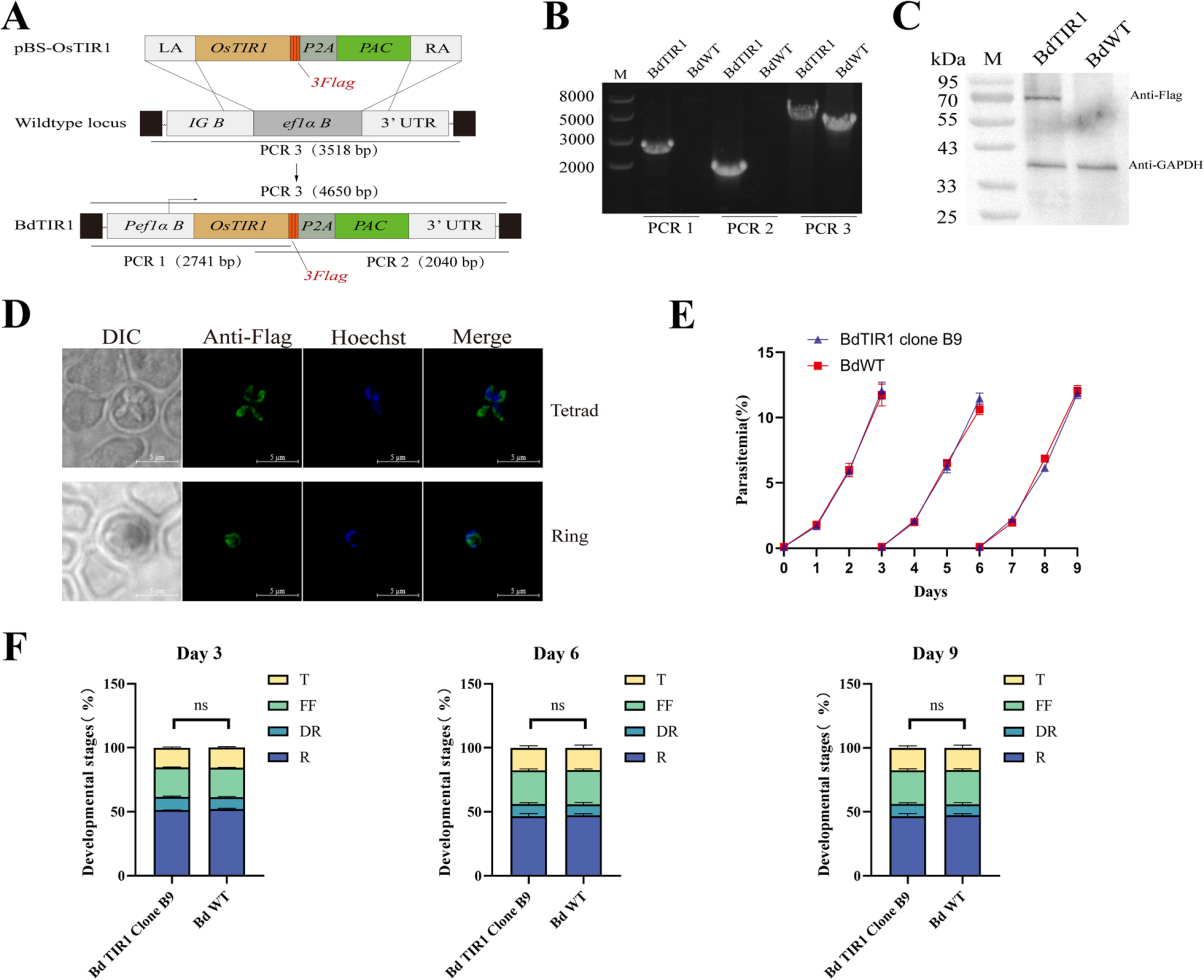
Generation of *B. duncani* strain stably expressing the OsTIR1 gene. **A** Schematic diagram illustrating the replacement of *ef-1αB* in the *B. duncani* WA1 strain with the auxin receptor OsTIR1 gene through homologous recombination, and PAC gene used as drug-selectable marker. PCR1, 2, and 3 denote the PCR for positive clone identification. **B** PCR on a BdTIR1 clone B9. **C** Western blot checking the expression of OsTIR1 in BdTIR1 B9 clone. OsTIR1 was detected by Flag antibody, whereas GAPDH was included as loading control. **D** IFA staining examining the location of OsTIR1 in BdTIR1 B9 clone. The differential interference contrast (DIC) image shows a parasitized RBC, Hoechst staining represents the nucleus of the parasite, and anti-Flag represents the location of OsTIR1. The merged image represents the overlap of Hoechst and anti-Flag channels. Scale bar = 5 μm.** E** The growth of BdTIR1 clone B9 was compared with the wild-type (WT) strain, and parasitemia was assessed daily by Giemsa-stained thin blood smears.** F** Percentages of different parasite forms of BdTIR1 clone B9 compared with WT. R, rings; DR, double rings; FF, filamentous forms, T, tetrads. Each value represents the mean result determined from at least three experiments including standard deviation values (SD); *ns* not significant, two-way analysis of variance (ANOVA)

To generate the plasmid pBS-OsTIR1 (Fig. 2A), a fragment of 658 base pairs (bp) upstream of the start codon and another 804 bp downstream of the stop codon of the *ef1αB* gene were amplified from *B. duncani* genomic DNA, serving as the homologous left and right arms, respectively. The OsTIR1 gene was obtained through sequence synthesis, and tagged with a triple Flag epitope sequence. The PAC gene was used as a puromycin-selectable marker, and a self-processing peptide P2A was placed between OsTIR1 and PAC to separate the two genes. The polymerase chain reaction (PCR) fragments were cloned separately into the *pBluescript* (pBS) backbone plasmid using the ClonExpress MultiS One Step Cloning Kit (Vazyme, China).

**Fig. 3:**
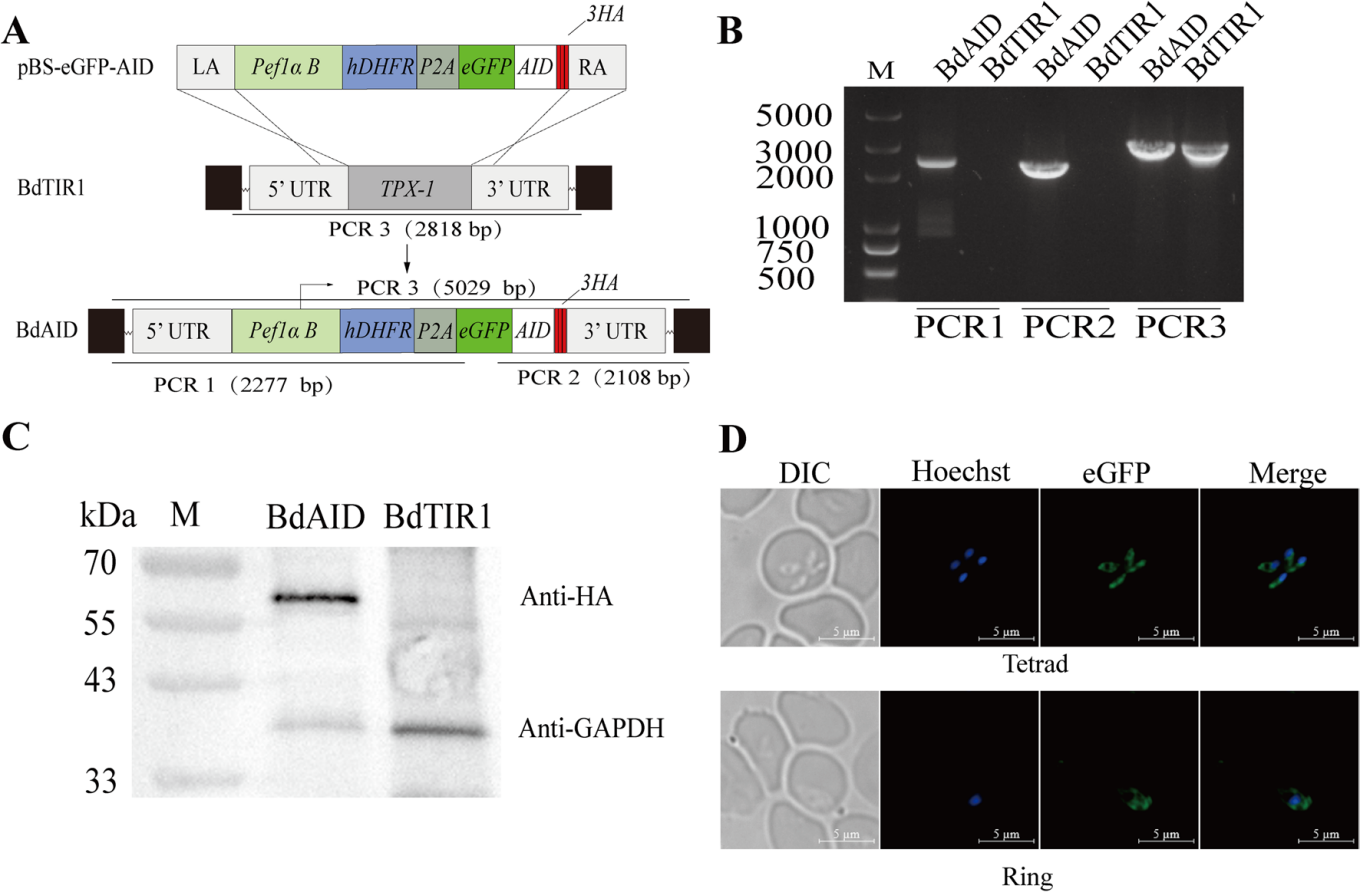
Generation of strain stably-expressing AID-tagged eGFP fused to 3×HA based on BdTIR1 clone B9. **A** Schematic diagram illustrating the replacement of *TPx-1* in the BdTIR1 clone B9 with the AID-tagged eGFP fused to 3×HA. PCR1 and 2 denote the PCRs for positive clone identification. **B** PCR on BdAID strain. **C** Western blot checking the expression of the eGFP-AID-3×HA fusion protein. The fusion protein was detected by HA antibody, whereas GAPDH was included as a loading control. **D** Live-cell fluorescence images examining the location of eGFP-AID-3×HA fusion protein. The merged image represents the overlap of Hoechst and eGFP channels. Scale bar = 5 μm

The plasmid pBS-eGFP-AID (Fig. 3A) was constructed following the same procedure as described above. Briefly, both 780-bp fragments upstream and downstream of the thioredoxin peroxidase 1 (*TPx-1*) gene were used as homologous arms, with the hDHFR gene serving as the drug screening marker for WR99210 resistance. The eGFP gene was combined with an AID degron and a triple HA epitope, and a self-processing peptide P2A was utilized to separate the drug-selectable marker from the eGFP-AID-3×HA fusion protein. All plasmids were sequenced to confirm the accuracy of their sequence.

### Transfection and drug selection

To generate transgenic parasites, *B. duncani* was handled according to the following protocol [21]: Infected RBCs (iRBCs) were washed twice with phosphate-buffered saline (PBS) and once with CytoMix buffer (120 mM KCl, 0.15 mM CaCl_2_, 10 mM K_2_HPO_4_, 10 mM KH_2_PO_4_, 25 mM HEPES, pH 7.6, 2 mM EGTA, and 5 mM MgCl_2_). A total of 50 μg of plasmid was dissolved in 100 μl of CytoMix buffer, thoroughly mixed with 100 μl of iRBCs, and the mixture was transferred to a 0.2 cm electroporation cuvette (Bio-Rad, Shanghai, China). Electroporation was then performed using a BTX electroporator at 1200 V, 25 mF, and two pulses. Twenty-four hours after the transfection, drug pressure (0.4 µg/ml puromycin or 5 nM WR99210) was initiated to select for correct transgenic parasites. Parasite genomic DNA was isolated using the TIANamp Genomic DNA Kit (TIANGEN, China) and used for PCR amplification. In addition, 5′ and 3′ integrations were confirmed by PCR using the specific primer pairs listed in Additional file 1: Table S1.

The method for obtaining monoclonal parasite strains follows the previously reported protocol [32, 33]: The culture was centrifuged to collect the pellet of iRBCs. A volume of 1 μl of iRBCs (~1 × 10^7^ cells) was then diluted 10,000-fold in culture medium. Subsequently, 1 μl of the final diluted mixture was added to 6 ml of culture medium containing 300 μl RBCs. After thorough mixing, 100 μl of the mixture was dispensed into each well of a 96-well plate. The culture medium was replaced every 3 days with 70 μl fresh medium until the parasites could be observed by light microscopy. When observing the parasites, PCR was used to identify the correct parasite strain [21].

### Susceptibility of parasites to auxin

To assess the influence of OsTIR1 protein expression on *B. duncani*, the transgenic parasite strain BdTIR1 was continuously cultured in vitro for three cycles (9 days) with initial parasitemia of 0.2% in a 96-well plate. Parasitemia and proportions of different forms of parasites were checked every 24 h by light microscopy examination of Giemsa-stained thin blood smears. To assess the toxicity of IAA to *B. duncani*, IAA (Sigma-Aldrich, I2886) was dissolved in 100% ethanol to prepare a 250 mM stock solution. Different concentrations of IAA (50, 100, 200, 400, 600,800, 1000, and 2000 μM) were added to the serum-free medium. The initial parasitemia was set at 0.1% to provide the parasites with more growth space. After continuous cultivation of *B. duncani* for 72 h, parasitemia was assessed. The data were analyzed using GraphPad Prism 8.3.0.

### Auxin induction assay of protein degradation

The transgenic parasite BdAID strain was cultured in a 48-well plate until the parasitemia reached 5–10%. The parasites were treated with IAA at final concentrations of 500 μM at different time points of 30 min, 1 h, 3 h, 5 h, 8 h, and 12 h before the sample collection. The control group received an equal volume of ethanol without IAA. When inducing with 2 mM IAA, the induction times were set to 30 min, 1 h, 3 h, 5 h, 8 h, and the culture continued for 12 h after 8-h induction (removal). The samples were washed with PBS to remove IAA and immediately collected and disposed for western blot and live-cell fluorescence microscopy.

### Western blot

To extract protein from *B. duncani*, iRBCs were washed with PBS to remove the culture supernatant and lysed with 0.1% saponin, followed by centrifugation to remove the hemoglobin. Next, 2× sodium dodecyl sulfate (SDS) loading buffer (0.2% bromophenol blue, 4% SDS, 100 mmol/l TrisHCI, pH 6.8, 200 mmol/l dithiothreitol [DTT], 20% glycerol) was added and incubated with the protein at boiled water bath for 10 min. The proteins were separated by 12.5% SDS–polyacrylamide gel electrophoresis (SDS-PAGE) and then transferred to polyvinylidene difluoride (PVDF) membranes (GE Healthcare, Shanghai, China), followed by incubation with blocking buffer (Tris-buffered saline with Tween 20 [TBST] with 5% skimmed milk) at room temperature for 1 h and then at 4°C overnight with anti-Flag antibody (rabbit; 1:5000; Proteintech, Shanghai, China) for detecting OsTIR1-3×Flag protein. Horseradish peroxidase (HRP)-conjugated goat anti-rabbit antibody (1:5000, Beyotime, Shanghai, China) was incubated with PVDF membranes for electrochemiluminescence (ECL) detection. The eGFP-AID-3×HA fusion protein was checked by western blot with anti-HA antibody (rabbit; 1:5000, ABclonal, Wuhan, China). The antibody of GAPDH (rabbit; 1:5000; Proteintech, Shanghai, China) was used as the loading control. The grayscale intensity of protein bands in western blot was quantified using ImageJ software [34].

### Fluorescence microscopy

For immunofluorescence assay (IFA), iRBCs with parasitemia of 5% were smeared onto slides and fixed with a mixture of 5% acetone and 95% methanol. After washing with fresh PBS, a 0.1% Triton X-100 solution was applied for 10 min. Subsequently, 3% bovine serum albumin (BSA) was used for blocking at 37 °C for 30 min and then 4 °C overnight with anti-Flag (rabbit, 1:200, Proteintech, Shanghai, China) to detect the location of OsTIR1 protein in *B. duncani*. Dylight 488 goat anti-rabbit immunoglobulin G (IgG) (1:1000, Abbkine, Wuhan, China) was incubated for 2 h at room temperature followed by a 10-min Hoechst 33342 (Sigma-Aldrich, Shanghai, China) incubation. After washing and air-drying, the samples were observed using a fluorescence microscope. For live-cell fluorescence, the iRBCs were washed twice with PBS to remove IAA and stained with 1 μg/ml Hoechst 33342 in PBS, and then fluorescence observation was conducted immediately. All images were captured using identical settings on an Olympus BX53 scanning microscope with a ×100 numerical aperture (NA, 1.20–1.40) oil objective; the images were processed using Zeiss ZEN 2.3 lite, and the fluorescence intensity was quantified using ImageJ [34].

## Results

### Identification of the homologs of the *B. duncani* SCF complex

In *B. duncani*, the AID system utilizes endogenous Skp1, Cul1, and Rbx1, in addition to transgenic OsTIR1 protein, to form the SCF complex. This complex recruits the AID degron-tagged eGFP when adding exogenous IAA, leading to eGFP ubiquitination followed by proteasomal degradation in *B. duncani* (Fig. 1A). The three endogenous proteins were identified in *B. duncani* as BdSkp1 (CNCB ID: GWHGBECJ000477, see Availability of data and materials), BdCul1 (CNCB ID: GWHPBECJ000337), and BdRbx1 (CNCB ID: GWHPBECJ001943) through conserved structure analysis and multiple protein sequence alignment. These three proteins in *B. duncani* shared identical domains with their counterparts in *O. sativa* (Fig. 1B). Specifically, the RING domain of Rbx1 binds to the C-terminal of the Cul1, forming the catalytic core of the ubiquitin-conjugating (E2) enzyme complex [35]. The Skp1 protein connects to the N-terminal of Cul1 and interacts with the F-box protein, collectively forming the SCF complex. This complex is regulated by neddylation of the Cul1 protein via its Nedd8 domain, impacting the complex formation and activity [36]. BdSkp1 and BdRbx1 exhibited significant sequence similarity to their orthologs in other apicomplexan parasites, yet demonstrated limited similarity to those in *O. sativa*. Specifically, BdSkp1 shared only 40.18% sequence identity with OsSkp1, while BdRbx1 shared 66.29% identity with OsRbx1, and Cul1 proteins exhibited generally low sequence similarity across these species, with BdCul1 showing just 21.16% identity shared with OsCul1 (Fig. 1C). Based on the reported crystal structure of the SCF complex [37–40], we identified and marked the key amino acid residues in the SCF complex of *B. duncani* essential for complex binding (Fig. 1C). In BdSkp1, three key residues, Asn 17, Asn 78, and Tyr 79, are highly conserved. Conversely, BdRbx1 exhibited a mutation at a key residue: Ile 7 (Phe in HsRbx1). The key residues in BdCul1 demonstrated low conservation compared to HsCul1, with only one out of six key residues being identical: Glu 33 (Met in HsCul1), Lys 36 (Tyr in HsCul1), Thr 47, Asp 40 (Tyr in HsCul1), Glu 128 (Tyr in HsCul1), and Gln 131 (Arg in HsCul1).

### Establishment of a strain stably expressing the OsTIR1 protein

To generate a *B. duncani* strain stably expressing OsTIR1, the gene needs to be inserted into a non-essential gene locus during the asexual stage. Previous studies have shown that *ef-1α* is a double-copy gene in *B. duncani*, and knocking out the *ef-1αB* gene does not affect the parasite growth [21]. The OsTIR1 gene with the drug-selectable marker was inserted into the *ef-1αB* locus by homologous recombination (Fig. 2A). A monoclonal strain, BdTIR1, stably expressing the OsTIR1 protein tagged with 3×Flag, was obtained through limited dilution. PCR1 and PCR2 confirmed the correct 5′ and 3′ insertion of the plasmid into the parasites' genomes. PCR3 of the BdTIR1 strain showed a different band from that of the WT, confirming the successful generation of a pure strain (Fig. 2B). The OsTIR1-3×Flag fusion protein had an expected molecular weight of 67 kDa, and western blot confirmed its expression in BdTIR1 strain, with no signal detected in WT (Fig. 2C). IFA also confirmed the expression of OsTIR1-3×Flag protein, and fluorescence could be observed in the cytoplasm of different forms of parasites (Fig. 2D). To assess the impact of the expression of OsTIR1 protein in *B. duncani*, parasitemia and different forms of parasites were recorded and analyzed daily. Throughout three consecutive cycles (9 days) of cultivation, no significant difference was observed in parasitemia between the BdTIR1 strain and WT (Fig. 2E). At the peak of parasitemia in each life cycle, where the numbers of different forms of parasites reached their maximum, the proportion of rings, double rings, filamentous forms, and tetrads in the BdTIR1 strain did not differ significantly from that in the WT (Fig. 2F). The results demonstrate successful integration of the OsTIR1 gene into the genome, with no significant impact on the growth and development of *B. duncani*.

### Construction of an AID degron-tagged eGFP strain based on the BdTIR1 strain

It has been demonstrated that *TPx-1* can be knocked out in both *B. bovis* and *B. duncani*, and the deletion of this gene does not affect the asexual blood stage of *Babesia* parasites [21, 41]. Therefore, inserting the eGFP reporter gene at this locus to validate the functionality of the AID system will not have a significant impact on *B. duncani*. The eGFP tagged with an AID degron and triple HA epitope was inserted into the *TPx-1* locus by homologous recombination, with the hDHFR gene used as the drug-selectable marker for resistance to WR99210 (Fig. 3A). Parasites were isolated from a single well in a 96-well plate using the limiting dilution method and identified by PCR. Parasite strains with the correct 5′ and 3′ integration of the plasmid into their genomes were confirmed by PCR1 and PCR2, validating the correct recombination into the *TPx-1* locus. However, PCR3 did not yield the expected 5029-bp band; instead, it showed a band identical to the WT (2818 bp). Even after changing the primers, the target band was still not amplified (Fig. 3B). Western blot detected a band corresponding to the eGFP-AID-3×HA fusion protein at its predicted molecular weight of 57 kDa in the BdAID strain, while no bands were observed in the BdTIR1 clone B9 strain (Fig. 3C). eGFP-AID-3×HA expression was also detected by live-cell fluorescence microscopy (Fig. 3D), and fluorescence could be observed in the cytoplasm of different forms of parasites.

These results indicate that the AID degron-tagged eGFP protein was successfully expressed in the parental BdTIR1 strain. All further experiments were conducted using this strain.

### Auxin-induced eGFP degradation in *B. duncani*

**Fig. 4:**
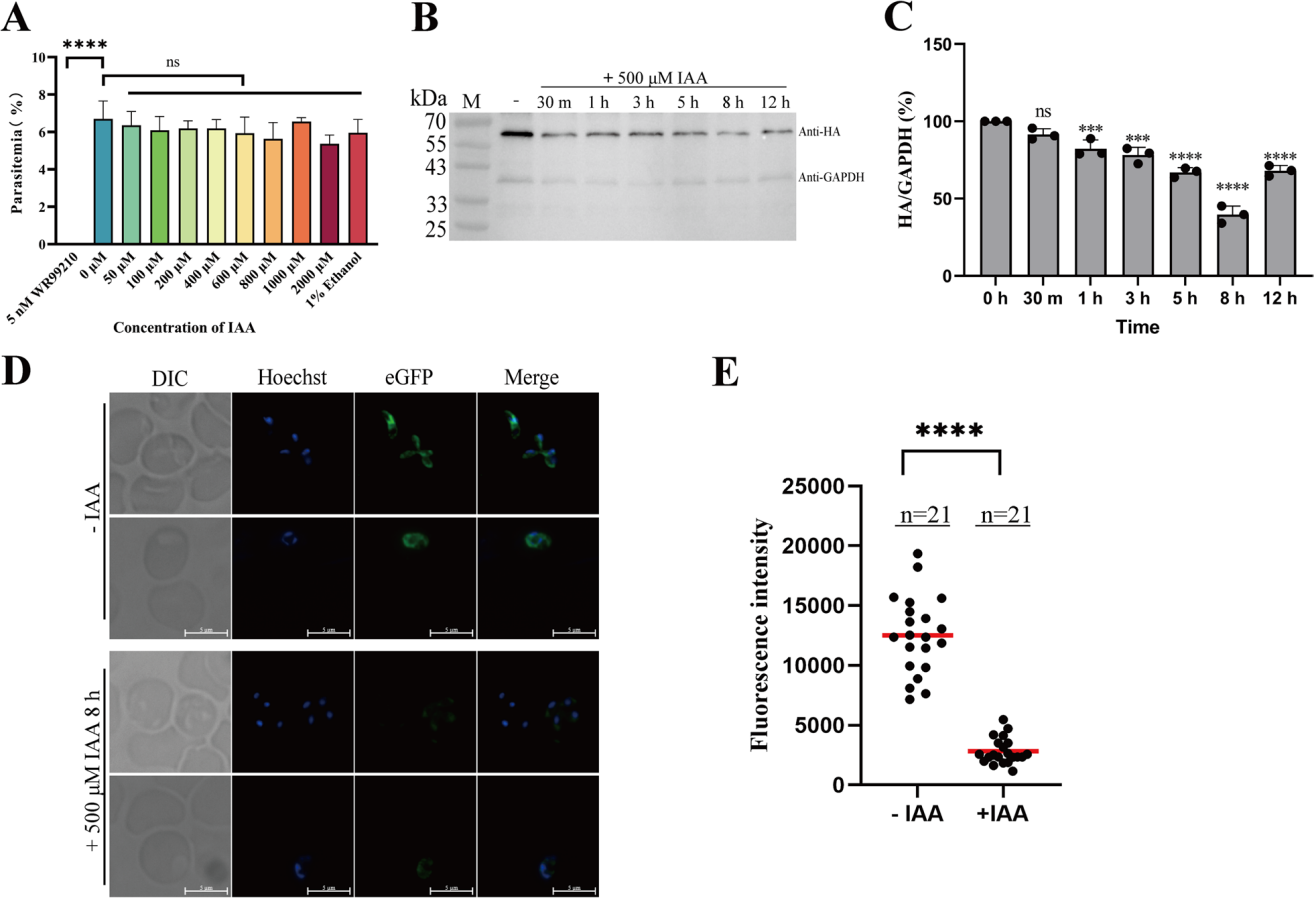
Auxin-induced eGFP degradation in *B. duncani*.** A** The impact of different concentrations of IAA on parasite growth. 1% ethanol was used as control to eliminate the influence of the solvent, whereas the ethanol percentage in the highest concentration IAA group was 0.8% (**** *P* < 0.0001, ns: not significant, one-way ANOVA; the error bars represent mean ± SD for three biological replicates). **B** Western blot checking the degradation of eGFP-AID-3×HA over time after 500 μM IAA treatment. The fusion protein was detected by HA antibody, whereas GAPDH was used as a loading control. **C** Grayscale values of the western blot bands. The grayscale values of eGFP-AID-3×HA were divided by the corresponding grayscale values of GAPDH at each time point, compared to the group without IAA for normalization (ns: not significant, *** *P* = 0.0009, *** *P* = 0.0001, **** *P* < 0.0001, **** *P* < 0.0001, **** *P* < 0.0001 **** *P* < 0.0001, one-way ANOVA; the error bars represent mean ± SD for three biological replicates). **D** Live-cell fluorescence images examining the expression level of eGFP-AID-3×HA fusion protein with or without 500 μM IAA treatment. The merged image represents the overlap of Hoechst and eGFP channels. Scale bar = 5 μm. **E** Fluorescence intensity of eGFP-AID-3×HA by fluorescence microscopy (*n* = 21, Student’s *t*-tests, **** *P* < 0.0001)

Before initiating the degradation of protein in *B. duncani* induced by IAA, the cytotoxicity of IAA was assessed, as it has shown cytotoxicity [42–45]. Continuous cultivation of *B. duncani* for 3 days under different concentrations of IAA (50, 100, 200, 400, 600,800, 1000, and 2000 μM) revealed that even at concentrations as high as 2 mM, IAA did not affect parasite growth (Fig. 4A). This result suggests that 500 μM IAA can be utilized for inducing the degradation of the target protein in *B. duncani*.

To assess the functionality of the AID system in *B. duncani*, parasites were treated with 500 μM IAA separately for varying time durations (30 min, 1 h, 3 h, 5 h, 8 h, and 12 h) to induce degradation, followed by collecting and disposing for western blot and live-cell fluorescence assays. Western blot analysis revealed that, compared to the group without IAA, there was partial degradation of eGFP-AID-3×HA upon the addition of IAA (Fig. 4B). Grayscale values for both HA and GAPDH in western blot were calculated separately, and by using GAPDH as loading control, the grayscale values of HA were normalized. The results (Fig. 4C) revealed that, compared to the group without IAA, there was no significant degradation induced by IAA within the initial 30 min, but apparent degradation was observed after 1 h of treatment, and the extent of degradation increased with the prolonged induction, reaching its maximum at 8 h, resulting in a 61.3% reduction of the target protein. However, the degradation diminished when treatment was prolonged to 12 h, possibly because proteasome-mediated protein degradation had reached saturation. The degradation of eGFP-AID-3×HA protein was also detected by live-cell fluorescence microscopy. The addition of IAA induced a significant reduction in the fluorescence intensity of eGFP (Fig. 4D), with an approximately 77.5% decrease (Fig. 4E) relative to the group without IAA.

**Fig. 5:**
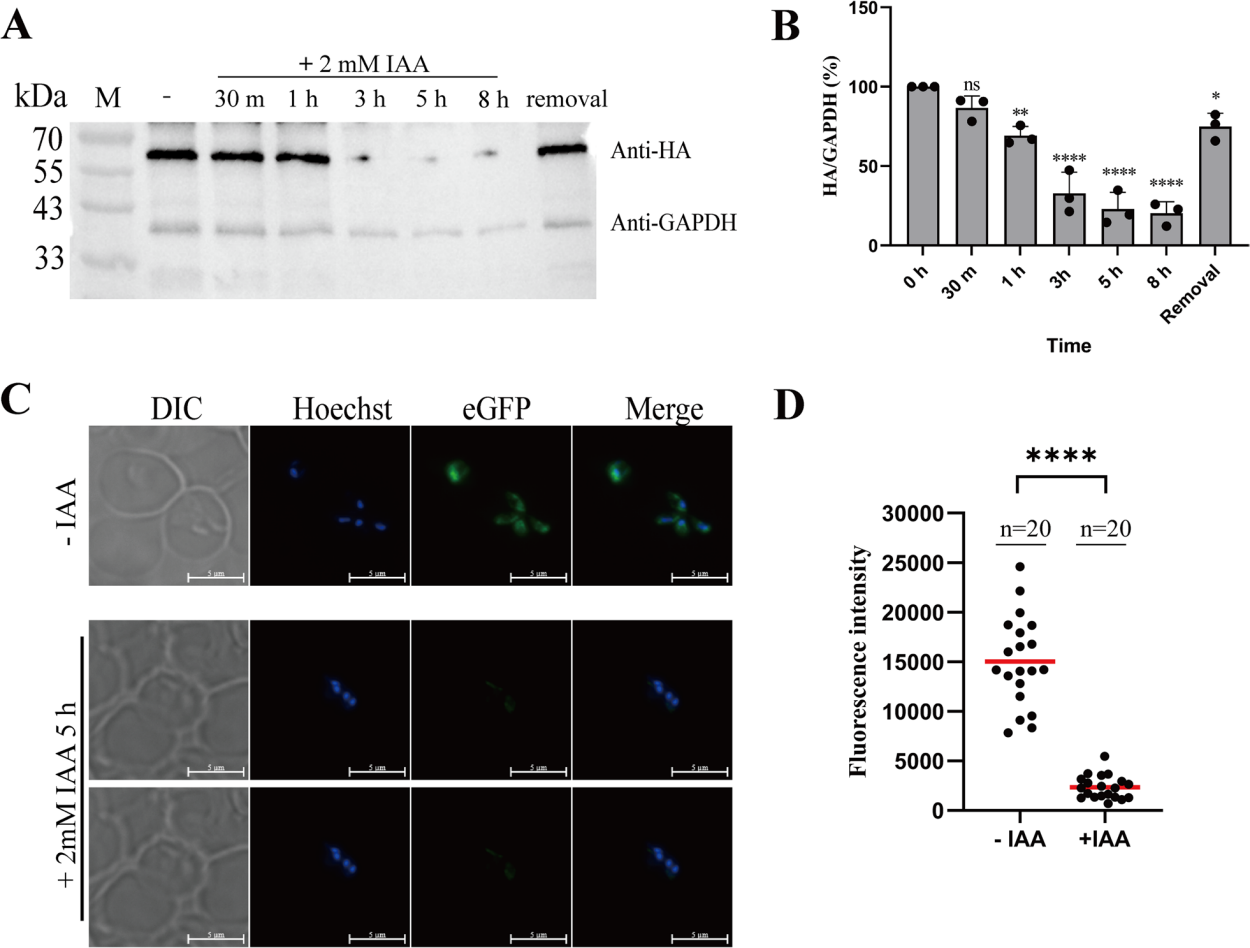
Increasing eGFP degradation by treatment of 2 mM IAA. **A** Western blot checking the degradation of eGFP-AID-3×HA over time after 2 mM IAA treatment. The "removal group" represents the treatment where 2mM IAA was used to induce degradation for 8 h, followed by the removal of IAA and an additional 12-h incubation. **B** Grayscale values of the western blot bands (ns: not significant, ** *P* = 0.0026, **** *P* < 0.0001, **** *P* < 0.0001, **** *P* < 0.0001, * *P* = 0.0127, one-way ANOVA; the error bars represent mean ± SD for three biological replicates). **C** Live-cell fluorescence images examining the expression level of eGFP-AID-3×HA fusion protein with or without 2 mM IAA treatment. The merged image represents the overlap of Hoechst and eGFP channels. Scale bar = 5 μm. **D** Fluorescence intensity of eGFP-AID-3×HA treated with or without 2 Mm IAA by fluorescence microscopy (*n* = 20, Student’s *t-tests*, **** *P* < 0.0001)

It was found that increasing the concentration of IAA to 2 mM could accelerate the degradation rate of eGFP, enhancing the protein degradation efficiency of the AID system (Fig. 5A). Gray value analysis indicated that treatment with 2 mM IAA for 3 h induced eGFP degradation to a comparable extent as 500 μM IAA did over 8 h. Moreover, after 5 h of treatment with 2 mM IAA, eGFP degradation reached a maximum level of 77% (Fig. 5B). The “removal” group represents the treatment with 2 mM IAA for 8 h followed by complete removal of IAA for 12 h to restore eGFP expression. The results showed partial recovery of eGFP, demonstrating the reversible protein degradation functionality of the AID system in *B. duncani*. Treatment with 2 mM IAA for 5 h demonstrated a greater reduction in eGFP fluorescence intensity than treatment with 500 μM IAA, showing an 84% decrease in the average fluorescence intensity of the parasites (Fig. 5C, D). These results indicate that AID-tagged target proteins could be degraded in the BdTIR1 strain, highlighting the functionality of the AID system in regulating protein levels in *B. duncani*.

## Discussion

Conditional knockdown systems are important for studying the function of essential genes, as they enable the regulation of gene expression at the DNA, RNA, and protein levels. The AID system is a highly efficient, rapid, and reversible method for regulating gene expression at the protein level. In the phylum Apicomplexa, the AID system was first established in *P. falciparum* [23], followed by *P. berghei* [24], *T. gondii* [26], and *P. yoelii* [25]. However, there were no reports of the AID system across the entire *Babesia* genus. Here, we constructed an AID system in *B. duncani*, providing an important method for studying gene function in this species.

The efficiency of protein degradation mediated by the AID system varies among different parasites in the phylum Apicomplexa, thereby limiting its application. Even within the same species, the efficiency of protein degradation varies when employing the AID system. In *T. gondii*, the degradation of calmodulin situated at the apical end of the cell is notably less efficient than that of yellow fluorescent protein (YFP) located in the cytoplasm [26, 46]. This disparity could be due to differences in the protein kinetics of different proteins, resulting in variations in their degradation rates. For the BdAID strain, we were unable to obtain a pure clone following the limiting dilution method (Fig. 3B). Strangely, PCR3 did not amplify the expected 5029-bp band for BdAID strain but rather a 2818-bp band identical to the WT. However, this strain was able to correctly express the eGFP-AID-3×HA fusion protein, and our research results demonstrated a decrease in eGFP expression levels detected by western blot and live-cell fluorescence microscopy after treatment with 500 μM IAA for 8 h. Therefore, we did not focus on the PCR3 issue with the BdAID strain and instead proceeded with the subsequent experiments. Furthermore, the results reveal that even after 12 h of treatment, the expression level of eGFP increased, potentially due to the parasite's adaptation to the effects of IAA or saturation of protein degradation mediated by the proteasome, resulting in the accumulation of newly synthesized proteins. Increasing the concentration of IAA to 2 mM accelerated the degradation of eGFP, and eGFP expression was restored after removing IAA for 12 h, demonstrating the reversible protein degradation functionality of the AID system. Although our results showed that continuous culture with 2 mM IAA for 72 h did not affect the growth of *B. duncani*, further studies are necessary to evaluate the potential impact of high IAA concentrations on knocking down endogenous genes. Based on the reported crystal structures of the SCF complex, we identified key amino acid residues involved in binding within the SCF complex of *B. duncani* that have undergone mutations. BdCul1 exhibits five mutated residues and showed low identity from other species. As Cul1 serves as the scaffold of the SCF complex, these mutations may affect the efficiency of protein degradation by the AID system in *B. duncani*.

The development of the AID system has greatly facilitated research into the function of essential genes, but this system has some limitations. The AID system has two main drawbacks: it requires high concentrations of 500 μM IAA to induce degradation, potentially leading to cellular toxicity and influencing the functionality of specific genes [42–45], and it exhibits leaky degradation even in the absence of IAA [43, 47–49]. Using OsTIR1 mutants along with specific ligands, such as OsTIR1 (F74G) with 5-Ph-IAA and OsTIR1 (F74A) with 5-Ad-IAA, demonstrates lower leaky degradation and lower ligand concentration than the conventional AID system [47, 50]. Utilizing F-box mutants and specific ligands enhances the efficiency of the system, while replacing F-box alongside AUX/IAA repressors is also a strategy to improve system efficiency. In mammalian cells, the use of *Arabidopsis thaliana* AFB2 (AtAFB2) as an alternative F-box protein instead of OsTIR1 was found to reduce leakage degradation, and substituting mAID degron with another AUX/IAA inhibitory factor, mini-AtIAA7 (37-104 aa), increased the kinetics of auxin-induced degradation [48, 51]. Another strategy to prevent leaky degradation is to tightly regulate the expression of OsTIR1. Using the doxycycline/tetracycline-inducible promoter to drive OsTIR1 expression partially mitigates basal degradation due to the efficiency of this system [52], while the competitive inhibitor of OsTIR1, auxinole, can effectively prevent this degradation and accelerate the reversible recovery of protein expression upon IAA removal [49]. These studies offer valuable strategies for improving the AID system, which will contribute to the future development of a more efficient system in *B. duncani*.

## Conclusion

 I﻿n this study, a transgenic monoclonal strain BdTIR1 expressing OsTIR1 was successfully constructed in *B. duncani*. This strain exhibited no significant difference from the WT in vitro and served as the parental strain for utilizing the AID system. Based on the BdTIR1 strain, the effective induction of eGFP degradation by 500 μM IAA, and the accelerated degradation at a concentration of 2 mM, demonstrates the crucial role of the AID system in regulating protein levels. Meanwhile, the efficiency of degradation by this system in *B. duncani* is lower than in other apicomplexan parasites and requires a high concentration of IAA, but it will still provide an important research tool for essential genes related to invasion, egress, and virulence, as well as a construction strategy for other apicomplexan parasites in which an AID system has not yet been established.

## Supplementary Information

Additional file 1: Table S1. Primers used in this study.

## Data Availability

The whole-genome sequence data for the *B. duncani* WA1 strain was obtained from the Genome Warehouse in the National Genomics Data Center, Beijing Institute of Genomics, Chinese Academy of Sciences/China National Center for Bioinformation (CNCB, https://www.cncb.ac.cn/), under accession number GWHBECJ00000000. Refseq of genomes of *B. duncani* used in this study: ef-1αB (CNCB ID: GWHPBECJ003656), TPx-1 (CNCB ID: GWHPBECJ003042), Skp1 (CNCB ID: GWHGBECJ000477), Cul1 (CNCB ID: GWHPBECJ000337), and Rbx1 (CNCB ID: GWHPBECJ001943).

## Abbreviations

eGFP: Enhanced green fluorescent protein
SDS-PAGE: Sodium dodecyl sulfate–polyacrylamide gel electrophoresis
TBST: Tris-buffered saline with Tween 20
PBS: Phosphate-buffered saline
HRP: Horseradish peroxidase
BSA: Bovine serum albumin
*TPx-1*: Thioredoxin peroxidase 1
YFP: Yellow fluorescent protein
