# Supplementary material for: Establishment of the auxin inducible degron system for Babesia duncani: a conditional knockdown tool to study precise protein regulation in Babesia spp
Source: Parasit Vectors. 2024 Oct 31;17:446. doi: 10.1186/s13071-024-06458-4 (PMC11526643; doi:10.1186/s13071-024-06458-4)
Supplement: Supplementary file 1 — Additional file 1: Table S1. Primers used in this study. [file 13071_2024_6458_MOESM1_ESM.docx]

**Additional file 1 Table S1.** Primers used in this study.

| **Table 1.** **Primers used in this study** | | |
| --- | --- | --- |
| **Primer** | **Sequence (5’ – 3’)** | **Use** |
| ef1 α-5H-R | TTCCTCAGGGAAGTAGGTCATTTTGTTCAA  TTGAAACTATATCCTTAGAA | To construct BdTIR1 plasmid |
| ef1 α-3H-F | cccgcaagcccggtgccTAAACTGAAATTACAATA  GAAACATTAACGC |  |
| TIR1-F | TGACCTACTTCCCTGAGGAAG |  |
| P2A-R | AGGACCGGGGTTTTCTTC |  |
| PAC-F | TGGAAGAAAACCCCGGTCCTaccgagtacaagcccacg |  |
| PAC-R | TTAggcaccgggcttgcggg |  |
| test-EF-1-T-F | GGTATTAATTCTAATTGTCTCCACG | PCR1 and PCR3 of BdTIR1 strain |
| OsTIR1-3Flag-R | CTTATCGTCATCGTCTTTGTAATCAATATCA | PCR1 of BdTIR1 strain |
| test-trans | CATCAATAATTTGCTGCTGCC | PCR2 and PCR3 of BdTIR1 strain |
| P2A-test | CAACTGCAGGAGCGAGAGAC | PCR2 of BdTIR1 strain |
| TPx-1-R | GTTTCTAATGACTAAAAATTAC | To construct BdAID plasmid |
| TPx-ef-f | GTAATTTTTAGTCATTAGAAACTTAGCAGTGGCACGAAAATTTC |  |
| hDHFR-R | gccgctgccCTGCTTatcattcttctcatatacttcaaat |  |
| P2A-F | AAGCAGggcagcggcGCCACGAACTTCTCTCTG |  |
| eGFP-R | CTAGCGCTCACCATCCTAGGCTTGTACAGCTCGTCCATGC |  |
| AID-3HA-F | CCTAGGATGGTGAGCGC |  |
| AID-3HA-R | TTAGGCATAATCTGGAACATCGT |  |
| TPx1-test-5f | TAGAGTTTGCAACTCCTGAAT | PCR1 of BdAID strain |
| EGFP-TEST-FR | CTGAACTTGTGGCCGTTTAC |  |
| TPx1-test-3R | TACAACCCGACGATTACAGT | PCR2 of BdAID strain |
| qPCR-eGFP-F | GAGCGCACCATCTTCTTCAA |  |
